# Outcome of chairside CAD/CAM ceramic restorations on endodontically treated posterior teeth: a prospective study

**DOI:** 10.1186/s12903-023-03812-3

**Published:** 2024-01-08

**Authors:** Su-Ning Hu, Jing-Wen Li, Xi-Xi Zhang, Rong Wei, Yu-Hong Liang

**Affiliations:** 1 Dental Clinic, Peking University International Hospital, Beijing, 102206 China; 2 Department of Cariology and Endodontology, Peking University School and Hospital of Stomatology, Beijing, 100081 China

**Keywords:** Outcome, CAD/CAM, Restorations, Endodontically treated posterior teeth

## Abstract

**Objective:**

The aim of this study was to evaluate the outcome and risk factors for chairside CAD/CAM full cusp coverage restorations on endodontically treated posterior teeth after 3 years of follow-up.

**Methods:**

A total of 245 endodontically treated posterior teeth of 224 patients were included and restored with CAD/CAM full cusp coverage all-ceramic restorations according to a standardized protocol. Patients were recalled after treatments 1 to 3 years and underwent clinical and radiological examinations. At recall, modified FDI criteria were used to determine treatment outcomes by 2 evaluators. Success was determined when FDI scores were 1–2, and failure was indicated when FDI scores were 5. Logistic regression analysis was performed to evaluate potential risk factors.

**Results:**

A total of 183 patients presented at recall, and the clinical outcomes of 201 teeth were analyzed with a recall rate of 82.0% for teeth and 81.7% for patients after 1–3 years of follow-up.185 of 201 teeth were found to have FDI scores of 1–2, and the success rate was 92%. No teeth were extracted during the follow-up period. Fourteen failed cases with an FDI score of 5 presented restoration dislocation, fracture of restoration or/and tooth. Logistic regression analysis revealed that oral parafunction (OR 2.281, 95% CI 2.2 ~ 47.5, *P* value 0.01) was a risk factor for success rate.

**Conclusion:**

Chairside CAD/CAM all-ceramic full cusp coverage restoration was (could be) a promising alternative for restoring endodontically treated posterior teeth.

## Introduction

Root canal treatment is a predictable choice to control endodontic-origin infection, and the reported success rate is up to 86-98% [1]. However, most endodontically treated teeth have suffered the loss of integrity of tooth structure and are vulnerable to fracture [1]. The endodontically treated posterior teeth with two-surface cavity preparation were resulted in a 46% loss in tooth stiffness and the teeth with MOD cavity resulted in a 63% lost [2]. In Nagasiri’s long-term research, tooth failure was identified in 101 teeth (45.9%) of 220 endodontically treated molars without crown coverage teeth at 5 years and the survival rate of the molars were 36% [3]. As the American Association of Endodontists (AAE) guidelines state, a full cuspal protective restoration could protect the remaining tooth structure and provide coronal sealing for endodontically treated posterior teeth. The full crown has been proved to protect the posterior teeth after endodontic treatment [4]. Aquilino and Caplan showed that endodontically treated teeth not crowned after obturation were lost at a 6.0 times greater rate than teeth crowned after obturation [5]. However, the crown preparation needs to remove an amount of tooth tissue, and the post core crown may increase the risk of root fracture and lateral perforation [6]. With development of bonding technology and further enhance of performance, indirect bonded ceramic restorations, such as inlays, onlays, overlays and endo-crowns are performed to restore endodontically treated posterior teeth [7], aiming to preserve more sound tooth structure [8].

In recent years, with the development of computer-aided design and computer-aided manufacturing (CAD/CAM) chairside techniques, restorations with these techniques have become a cost-effective alternative for endodontically treated posterior teeth and popular in practice. Plenty of studies have evaluated the longevity and survival rate of ceramic onlay restoration which covers all the tooth cusps. Strasding evaluated the survival rate and the technical and biologic outcomes of all-ceramic onlay restorations in premolars and molars and indicated the overall 11-year survival rate of the onlay restorations was 80.0% [9]. And in study of Irusa showed the survival rate of ceramic onlay restorations were 81.1% for 6–22 years [10]. However, there is limited information available regarding the outcome and factors influencing the outcome of CAD/CAM onlay restorations [11]. Therefore, this study aimed to observe and evaluate the clinical performance of chairside CAD/CAM ceramic onlays which covered all the cusps with cavity retention on endodontically treated posterior teeth and identify the potential influencing factors.

## Materials and methods

### Inclusion and exclusion criteria

A total of 224 patients who received endodontic treatment and chairside CAD/CAM full cusp coverage restoration in the Department of Dental Clinic, Peking University International Hospital, China, were consecutively recalled after 1 to 3 years. All the patients included in the study met the following criteria:

1. Endodontically treated posterior teeth were free of symptoms;
2. Tooth defects involving no more than 3 tooth surfaces and all margins above the gingiva;
3. Presence of the opposite tooth and at least one proximal tooth.

Patients with active periodontal disease, malocclusion, definite parafunctional habits, and microfracture teeth were excluded. The study was approved by the Ethics Committee of Peking University International Hospital (No. 2018-032 < BMR>), and all included patients were required to sign written informed consent.

### Clinical procedure

A total of 245 ceramic restorations manufactured by using the CEREC SW4.5.2 chair-side system was placed in 224 patients (97 males and 127 females, average age 37.7 years). The treatments were carried out by 3 endodontists and 4 prosthodontists according to standard procedures as follows.

Clinical and radiological examinations were performed, and preoperative factors, such as tooth type, sex, jaw, age, oral hygiene (calculus index 0–3), chewing habits (bilateral and unilateral), occlusal wear (tooth wear index 0–3) and oral parafunction were recorded. The pulp chamber was built up by composite resin. The tooth was prepared, and the cavity was created with a flat floor and a slightly divergent tap of 8 to 10 degrees, leaving no undercut. All the cusps were covered by restorations and were reduced according to anatomical form at least 1.5-2 mm. All the internal angles were rounded and polished. The depth of the cavity, the width of the remaining walls and the position of the contact area were recorded.

Preparations, adjacent and opposite teeth were scanned directly with a digital scanner (CEREC Omnicam, Sirona, Germany). Restorations were designed by a technician using Cerec Software 4.4.4 (CEREC, Sirona, Germany) and fabricated with ceramic blocks (IPS e.max CAD, Ivoclar Vivadent, Principality of Liechtenstein) using a dental CAD/CAM milling machine (CEREC MC X, Sirona, Germany). All laboratory procedures for the restorations were performed in strict adherence to the manufacturer’s instructions.

At the try-in session, marginal adaptation, contact area, and color were examined. The restorations covered with adhesive cement (Multilink, Ivoclar Vivadent AG, Principality of Liechtenstein) were placed on prepared teeth according to the manufacturer’s instructions.

### Assessment

the restorations were evaluated after treatments for 1 to 3 years, by two independent evaluators who were prosthodontists with five years of experience according to modified FDI criteria [13]; patient satisfaction was also investigated. According to modified FDI criteria, FDI level 1–2 was defined as success, and level 5 indicated failure. When there were disagreements in evaluations between the evaluators, the worse outcome was adopted. Photos and periapical radiographs were taken preoperatively and postoperatively. The failed restorations that could not function were replaced by a new restoration. The included patients who refused recall were enquired by phone about the restoration.

### Statistical analysis

The statistical analyses were calculated by SPSS (IBM SPSS Statistics 20). The response bias between recalled and dropout cases was analyzed by the chi-square test. The Kappa test was performed to evaluate intra- and inter-examiner agreement. Multivariate logistic regression analysis was performed to identify prognostic factors. The level of significance was set to 0.5.

## Result

### Information on recall

There were 183 out of the 224 included patients, aged 21 to 72 years (mean 38 years), with 201 all-ceramic restorations presented at recall after treatments from 1 to 3 years (median 18 months). The recall rate was 82.0% (201/245) for teeth and 81.7% (183/224) for patients. The reasons for dropout included that patients were unable to contact or refused to recall. There was no significant difference between the dropout and recalled cases in clinical factors *(P > 0.05) (*Table 1*)*.

### Outcome assessment and prognostic factor analysis

**Fig. 1:**
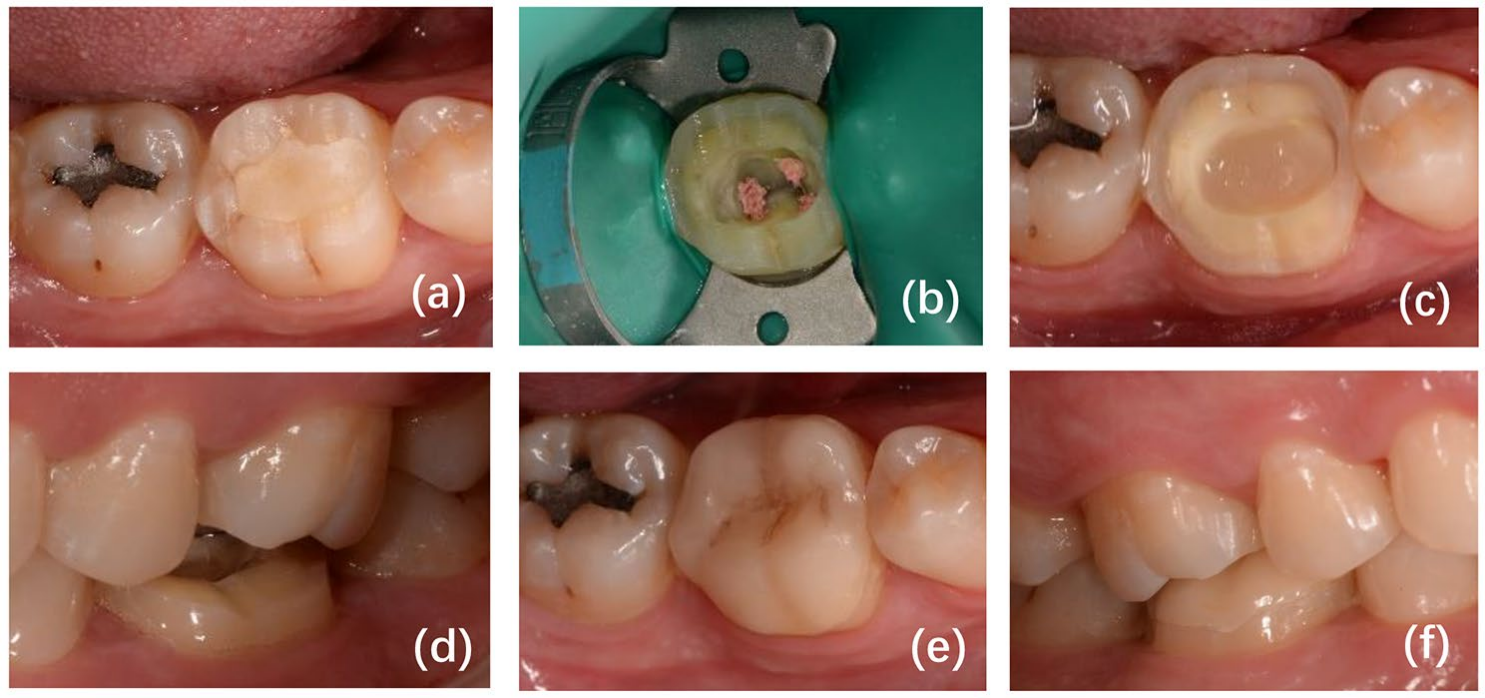
A left mandibular first molar of a 35-year-old male who received endodontic treatment **(a)**. After the examination, we decided to restore the molar with a ceramic restoration covering all cusps **(b-d)**. The ceramic restoration was placed and bonded with resin adhesive **(e)**. After 27 months, the patient was recalled, and the restoration was evaluated by the FDI and received a score of 5 **(f)**

**Fig. 2:**
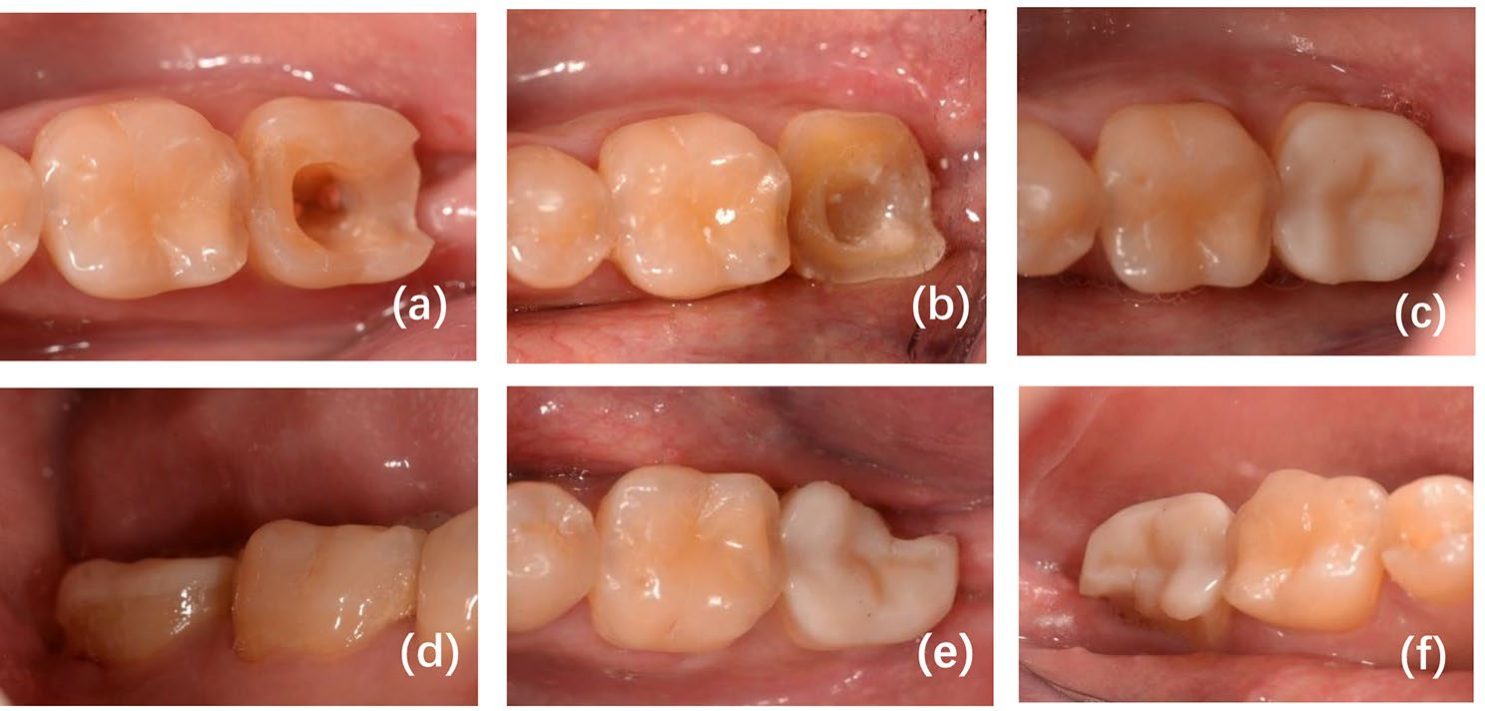
A second molar in the mandible of a 35-year-old male received root canal treatment **(a)** and was prepared with the principles of the ceramic full cusp coverage restoration **(b)**. The restoration was placed and determined to be successful according to FDI criteria **(c-d)**. However, the restoration and tooth were both fractured in a 6-month recall assessment **(e-f)**. The restoration was removed and replaced by a ceramic crown

The inter-examiner Kappa value in determining the outcome of restorations was 0.785. The intra-examiner values were 0.918 and 0.865, respectively. At recall, 185 f 201 teeth with FDI levels 1–2 were categorized as successful (92%) (Table 2*) (*Fig. 1*)*. The patient satisfaction rate was considered 98%. No teeth had been extracted during the follow-up period. Fourteen of 201 restorations (7.0%) were considered failures *(*Table 3*) (*Fig. 2*)*. Eleven of 14 failure cases (78.6%) were due to dislocation of restorations necessitating re-cementation after 9 to 39 months of service. Two fractured restorations (14.3%) required the procedure to be redone. The remaining molar experienced root fracture and underwent root resection surgery. No patient experienced failure of two or more restorations.

The bivariate analysis for the effects of clinical factors on dichotomous outcome was summarized in Table 4. Logistic regression analysis revealed that oral parafunction influenced the treatment outcome significantly (OR 2.281, 95% CI 2.2 ~ 47.5, *P* value 0.01).

## Discussion

In this study, the success rate of restorations on endodontically treated posterior teeth was 92.0% in 9–39 months (median 18 months). A review by Jaafar Abduo reported that ceramic restorations had a cumulative survival rate of 91-100% in 2–5 years and 71-98.5% in more than 5 years [12], which was consistent with the findings of our study. The recall rate is important for outcome studies. In the present study, 44 teeth from 41 patients were not available for follow-up. For the dropout patients who refused to be recalled to the hospital, telephone recall was performed to acquire more information on outcomes and to minimize the effect of dropouts. All the teeth from the dropout patients were functional. There were 15 follow-up participants included two teeth, and one participant with four teeth. All these restorations were successful, so they weren’t analyzed separately.

To evaluate the success rate of all-covered cusp restorations placed on endodontically treated posterior teeth, modified FDI criteria were used. The modified United States Public Health Service (USPHS) was the most commonly used criterion for the clinical assessment of dental restorations. However, Hickel et al. proposed a more sensitive and discriminative scale in 2007 that was based on aesthetics, function and biology to detect early deterioration and signs of failure [13]. These criteria were considered “Standard Criteria” by the Science Committee of the FDI World Dental Federation in 2007 [14]. Each category was divided into 16 subcategories, and each subcategory was scored by 5 levels. Scores of 1–3 indicated “acceptable restoration”, and scores of 4–5 suggested failure. In this study, 12 of 16 subcategories that were suitable to evaluate CAD/CAM ceramic restorations were adopted, and the final score of each restoration was determined by the worst score among all subcategories.

In this study, 11 of 14 failed restorations (in 6 men and 5 women; 6 premolars and 5 molars) were deboned, and 2 (2 men, 1 premolar and 1 molar) had fractures in the restorations and/or teeth. Jaafar Abduo [15] summarized the factors affecting the longevity of ceramic onlays (all-covered cusp restoration), which included the thickness of the restoration, fabrication materials and methods, restoration location, bonding and cementation agent, tooth vitality and parafunctional habits. Previous studies reported that molar onlays were 3–4 times more likely to fail than premolar onlays [16, 17]. However, there was no significant difference in the success of ceramic restorations in this study, which is consistent with the findings of the study by Beier [17]. Secondary caries was reported as a cause of restoration failure by several studies, in which 6.3–40.0% of all failures were due to caries [17–20]. In our study, secondary caries in 10 restorations were detected, and 7 of the 10 onlays were premolars. Premolars were at 5.485 times higher risk of secondary caries than molars (*P* = 0.016, OR = 5.485). Furthermore, the margins of 4 restorations with secondary caries were placed at the proximal surface. We presumed that there was no good approximal fit to prevent plaque accumulation that may lead to secondary caries [21].

In the present study, oral parafunction was the only factor influencing the longevity of restorations. Oral parafunctions included nonfunctional gnashing, bruxism, clenching of teeth, and habits including but not limited to nail biting, chewing on cheeks or other mucosa, and chewing on pens or other objects that could affect the stomatognathic system [22]. Several studies have shown a negative effect of parafunctional habits on restoration longevity. Studies by Smales reported that patients with parafunctional habits had a greater chance of restoration failure [23, 24]. In some studies, patients with parafunctional habits were excluded [18, 25, 26], as was the case in our study. However, it was difficult to make dentists and patients aware of bruxism. Thorough and careful examination should be carried out to identify the potential greater risk of parafunctional habits [22].

However, a long-term study is required to observe the stability of onlay restorations. Besides, additional studies are also needed to compare the effects between onlay restorations and crowns, and exploring which type of onlay restorations are more appropriate for tooth defect.

## Conclusion

Based on the present study observations, chairside CAD/CAM ceramic restorations could provide a promising alternative to restore endodontically treated posterior teeth, and oral parafunction negatively influences the outcome of restorations.

**Table 1:** Analysis of clinical factors in the reviewed (N = 201) and dropout cases (N = 44)

Factors	Reviewed group (%)		Dropout group (%)	*P* value
Tooth type				0.276
premolar	64 (31.8)		11 (25)	
molar	137 (68.2)		33 (75)	
Sex				0.639
male	90 (44.8)		15 (34.1)	
female	111 (55.2)		29 (65.9)	
Maxilla vs. Mandible				0.651
maxilla	90 (44.8)		17 (38.6)	
mandible	111 (55.2)		27 (61.4)	
Age				0.371
≤ 34 years	78 (38.8)		24 (54.5)	
> 34 years	123 (61.2)		20 (55.5)	
Oral hygiene				0.062
good	95 (47.3)		14 (31.8)	
poor	106 (52.7)		30 (68.2)	
Chewing habits				0.732
bilateral	160 (79.6)		34 (77.2)	
unilateral	41 (20.4)		10 (22.8)	
Oral parafunction				0.864
presence	8 (4.0)		0 (0)	
absence	193 (96)		44 (100)	
Occlusal wear				0.848
presence	111 (55.2)		19 (43.2)	
absence	90 (44.8)		25 (56.8)	
Total	201		44	

**Table 2:** Quality of ceramic restorations based on modified FDI criteria

Criteria	Level 1	Level 2	Level 3		Level 4	Level 5
Surface luster	197	4		0	0	0
Staining (surface, margins)	159	42		0	0	0
Color match and translucency	147	54		0	0	0
Esthetic anatomical form	196	5		0	0	0
Marginal adaptation	116	85		0	0	0
Occlusal contour and wear	200	1		0	0	0
Fracture of restorative material and restoration retention	177	8		3	0	13
Approximal contact point and food impaction	157	44		0	0	0
Radiographic examination	176	25		0	0	0
Recurrence of caries (CAR), erosion, abfraction	191	10		0	0	0
Tooth integrity (enamel cracks, tooth fractures)	197	3		0	0	1
Periodontal response (always compared to a reference tooth)	178	23		0	0	0
Adjacent mucosa	199	2		0	0	0
Oral and general health	197	4		0	0	0

*In all the categories of FDI criteria, the sum of the lowest percentages for Levels 1–2 were considered as the success rate and that for Levels 1–4 was considered as the survival rate

**Table 3:** Analysis of failed ceramic restorations (N = 14)

No.	Failure	Survival Time (m)		No.	Failure	Survival Time (m)
1	Restoration dislocation	9		8	Restoration dislocation	24
2	Fracture of root	10		9	Restoration dislocation	26
3	Restoration dislocation	11		10	Restoration dislocation	26
4	Restoration dislocation	11		11	Fracture of restoration and secondary caries	27
5	Restoration dislocation	20		12	Fracture of restoration	29
6	Restoration dislocation	21		13	Restoration dislocation and secondary caries	22
7	Restoration dislocation and secondary caries	23		14	Restoration dislocation	34

**Table 4:** Summary information of clinical factors influencing the survival rate of restorations

Factors	No.	Survival restoration (%)
Tooth type		
premolar	57	89.1
molar	130	94.9
Sex		
male	81	90.0
female	106	95.5
Jaw		
maxilla	83	92.2
mandible	104	93.7
Age		
≤ 34 years	75	96.2
> 34 years	112	91.1
Oral hygiene		
good	90	94.7
poor	97	91.5
Chewing habits		
bilateral	149	93.1
unilateral	38	92.7
Oral parafunction		
presence	5	62.5
absence	182	94.3
Occlusal wear		
presence	105	94.6
absence	82	91.1
Total	187	93

## Data Availability

The datasets used and/or analyzed during the current study available from the corresponding author on reasonable request.
